# Model-building strategies for low-resolution X-ray crystallographic data

**DOI:** 10.1107/S0907444908040006

**Published:** 2009-01-20

**Authors:** Anjum M. Karmali, Tom L. Blundell, Nicholas Furnham

**Affiliations:** a Department of Biochemistry, University of Cambridge, 80 Tennis Court Road, Cambridge CB2 1GA, England

**Keywords:** model building, low-resolution data

## Abstract

The interpretation of low-resolution X-ray crystallographic data proves to be challenging even for the most experienced crystallographer. Ambiguity in the electron-density map makes main-chain tracing and side-chain assignment difficult. However, the number of structures solved at resolutions poorer than 3.5 Å is growing rapidly and the structures are often of high biological interest and importance. Here, the challenges faced in electron-density interpretation, the strategies that have been employed to overcome them and developments to automate the process are reviewed. The methods employed in model generation from electron microscopy, which share many of the same challenges in providing high-confidence models of macromolecular structures and assemblies, are also considered.

## Abstract 2

Interpretation of low-resolution X-ray crystallographic data can prove to be a difficult task. The challenges faced in electron-density interpretation, the strategies that have been employed to overcome them and developments to automate the process are reviewed.

## 1. Introduction

**Figure 1:**
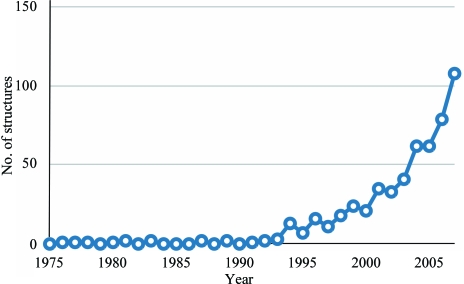
Rate of deposition of low-resolution structures: the number of new structures deposited each year in the Protein Data Bank which were solved by X-ray crystallographic methods at a resolution of less than 3.5 Å.

The number of low-resolution structures solved by X-ray crystallography being deposited in the Protein Data Bank (PDB; Berman *et al.*, 2000 ▶) has rapidly increased in recent years (see Fig. 1 ▶). Whereas previously such data sets may have been discarded in pursuit of higher resolution, the value of the biological information that can only be obtained from lower resolution data has begun to be realised. It has been observed that the threshold of acceptability for obtaining mechanistic insights has been changing (Brunger, 2005 ▶), with a number of significant structures, such as the entire ribosome (at 7 Å resolution; Cate *et al.*, 1999 ▶), plant photosystem I (at 4.4 Å resolution; Ben-Shem *et al.*, 2003 ▶), the reverse transcriptase from HIV in complex with a target RNA (at 4.7 Å resolution; Jaeger *et al.*, 1998 ▶) and many others being described. The increased reporting of low-resolution structures has coincided with advances in both experimental and computational methods for structure determination that make tackling the problems associated with low-resolution data increasingly tractable.

In this review, we provide an overview of the techniques used in building a model from low-resolution electron-density maps. This can be one of the most time-consuming, laborious and difficult tasks in the structure-determination process. There are a number of techniques in data processing and refinement, which will only be briefly mentioned, that can greatly aid the structure-determination process. Current refinement techniques have been reviewed by DeLaBarre & Brunger (2006 ▶) and elsewhere in this issue. The model-building methods applied to X-ray crystallography are contrasted with those used in modelling electron-microscopy data and the difference in definitions of resolution between the two methods is examined.

## 2. Problems of interpreting low-resolution X-ray data

**Figure 2:**
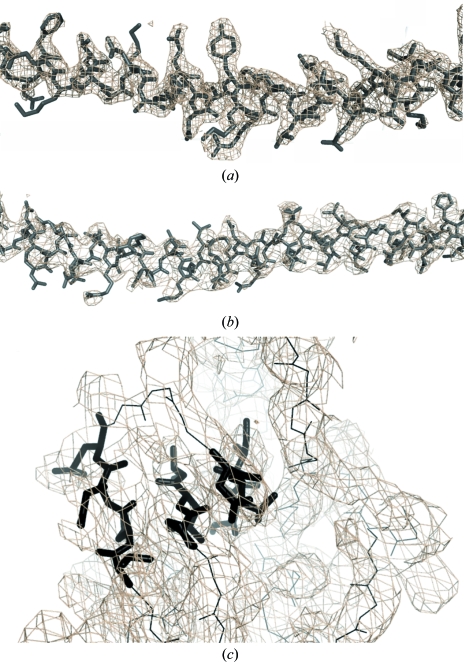
Features of high- and low-resolution electron-density maps. (*a*) A section of the high-resolution structure of XRCC4 solved at 2.3 Å resolution. (*b*) The equivalent section to (*a*) from the low-resolution structure of Lif1 solved at 3.9 Å resolution. Note the loss of side-chain and main-chain features. (*c*) A section of β-sheet from the 3.9 Å resolution structure of Lig4. The region of β-sheet is shown as black sticks, while the remaining trace is depicted as black lines. In all three the maps are calculated with 2*F*_o_ − *F*_c_ coefficients and thus may have some model bias.

A number of factors contribute to the problem of generating an atomic model from low-resolution (*d* > 3.5 Å) X-ray diffraction data. The primary cause of difficulty is that the number of observations used in the calculation of the electron-density map is significantly smaller than the number of parameters to be defined. This results in a map with a lack of atomicity, with helices appearing as tubes of density, lack of definition of peptide groups and accumulation of density in places other than the main chain (see Fig. 2 ▶). Furthermore, termination of the Fourier series at low resolution can cause diffraction ripples around peaks in electron density, making the map difficult to interpret.

Faced with such problems, the crystallographer has difficulty in tracing the peptide main chain, with ambiguities in direction and in the number of residues that make up sections of the structure. For example, the conformations of residues that cap a helix are often unclear as the helix unwinds into a turn region. In addition, it is also difficult to ascertain the number of residues that then make up the loop. Once a main-chain trace (or part trace) has been constructed, the assignment of residue type, through the placement of its side chain, is also taxing. Registry errors can easily occur as density for long side chains can be curtailed owing to side-chain disorder, resulting in the assignment of a residue with a shorter side chain instead. Further problems can arise with bulkier side chains, for example distinguishing between phenylalanine and tyrosine, where the lack of atomicity can result in the tyrosine hydroxy group being indiscernible. Assignment can also be hampered by the fragmentation of the main-chain trace, such that even if a section can be correctly assigned it cannot be continued for the entire structure. The more discontinuity in the main-chain trace, the more intractable the sequence assignment becomes.

## 3. Classical and current strategies for model building into low-resolution electron density

The issues of handling low-resolution electron density have been present since the first protein structures were experimentally determined. The early structure of myoglobin by Kendrew and coworkers in 1958 was determined at 6 Å resolution (although subsequently to 2 Å). It is interesting to note that although the structure was solved at 6 Å resolution the crystal actually diffracted to a much higher resolution, but owing to problems in processing all the reflections the resolution was cut back (Kendrew *et al.*, 1958 ▶). The model was built as a tube connecting continuous peaks in electron density plotted on stacked sheets of glass (Kendrew, 1958 ▶). There were ambiguities in the trace, with at least two ways of tracing the molecule, which were not resolved until the 2 Å resolution model was built using the now-famous Kendrew wire models. Yet the 6 Å resolution model provided a rich resource of new insights into protein structure. Similar problems were en­countered in the structure of haemoglobin solved by Perutz and coworkers at 5.5 Å resolution a short while after. The model was constructed by cutting, from a sheet of plastic, the shape of each peak above a certain cutoff and then assembling the pieces according to their positions in the different sections (Muirhead & Perutz, 1963 ▶).

Unlike these early model-building methods that relied just on the electron density, today there is a large knowledge base for macromolecular structure from macromolecular studies at high resolution as well as detailed analysis of small peptides. Often, there are fragments or domains of the structure under study, or at least a structural homologue, that have been solved at high resolution. These fragments can be used to perform molecular replacement. If these sections are too small or structurally dissimilar to be used for phasing, they can be manipulated manually within a graphics-based modelling program [such as *O* (Jones *et al.*, 1991 ▶) or *Coot* (Emsley & Cowtan, 2004 ▶)] to obtain an approximate orientation of the section in the electron density. If fragments of previously determined structures are unavailable, sections of idealized secondary-structure fragments can be used. This can be particularly useful for α-helices as even at low resolution the groove of the helix can be discerned in the electron density. These fragments can then be connected by manually extending the main-chain trace using tools such as the ‘baton’ tool in *Coot*, which has a defined residue length to place C^α^ atoms at appropriate distances apart. This is often performed in conjunction with an alignment of the sequence of the structure under investigation with structural homologues (supplemented with other homologous sequences to improve the alignment accuracy). From the sequence/structure alignment, secondary-structure elements can be inferred and the number of residues separating them can be estimated.

The above general strategy was broadly employed in solving the structure of plant photosystem I at 4.4 Å resolution. The crystallo­graphers utilized the C^α^ backbone of a subsection of a previously solved homologue, the cyanobacterial reaction centre, which was manually located in the electron density. This provided a core to which modifications (residue additions/deletions) could be made based on clear parts of the map and in combination with a sequence alignment. Idealized helices were also placed and manually modified to improve their fit to the map. Further subunits were assigned based on biochemical and other biophysical data, although the entire model could only be represented as a backbone trace (Ben-Shem *et al.*, 2003 ▶). A similar backbone-only trace was generated for the first structural model of the bovine mitochondrial F_1_-ATPase (at 6.5 Å resolution) using a tracing program (‘skeletonization’ in *O*, which reduces the electron density to idealized thin lines following the long polypeptide chains preserving the connectivity of the structure; Greer, 1985 ▶). At this resolution the automated methods make many misinterpretations; thus, the trace was manually edited to exclude all atoms placed outside the density (Abrahams *et al.*, 1993 ▶). This structure was subsequently solved at 2.7 Å resolution (Abrahams *et al.*, 1994 ▶). A stripped-down polyglycine version of this higher resolution model was used as a molecular-replacement probe to determine the structure of the *Escherichia coli* mitochondrial F_1_-ATPase at 4.4 Å resolution. Further manual modelling to account for differences between the search model and the electron density, including extending into new regions, was conducted. Side-chain modelling was not possible and the model was deposited as a polyglycine model (Hausrath *et al.*, 1999 ▶).

While tracing the main chain can be challenging, the modelling of side chains can be even more problematic. A C^α^ trace may be all that can be confidently modelled, unless there are clear features in the electron density that can be used as points to begin to assign sequence. Features can include large ‘blobs’ that can be attributed to a large side chain, most commonly tryptophan, combined with topological features seen in related structures, which might indicate relationships to other secondary structures. In addition, unusual topological features produced by sequence motifs can also aid in assigning sequence. Most useful are peaks in the density from heavy atoms used in phasing from MAD, SAD or MIRAS experiments. Sequence can also be attributed by extension from a fragment of a high-resolution structure if one has been docked or used for molecular replacement. Other modifications present in the structure, such as glycosylation sites or disulfide bridges, are also invaluable in acting as sequence-anchor points. Often, combinations of features are required to assign sequence effectively.

In the case of the 30S subunit (solved at 5.5 Å resolution), seven high-resolution structures were placed manually using both visual interpretation and other extensive experimental data including a neutron map of the centres of mass, footprinting studies and accumulated biochemical data. Un­determined substructures have also been resolved on the basis of helical secondary-structure predictions from biochemical and neutron scattering data as well as one section based on a secondary-structure prediction from the sequence (Clemons *et al.*, 1999 ▶). In determining the 50S subunit (solved at 5.0 Å resolution), in addition to the placement of previously solved fragments, sections were identified using template fragments placed using *ESSENS* (Kleywegt & Jones, 1997*a* ▶) and some unusual shapes, *e.g.* the sarcin–ricin loop with a distinctive S shape. This became a marker to orientate other sections, such as the L6f region (Ban *et al.*, 1999 ▶). In determining the entire 70S ribosome at 7.8 Å resolution, similar methods of com­bining molecular replacement, in this case using a pseudo-atom model from an EM single-particle reconstruction, in combination with inferences from biochemical data as well as knowledge of the 30S and 50S components was used to generate an all-atom model (Cate, 2001 ▶; Cate *et al.*, 1999 ▶).

In determining the structure of the fully glycosolated SIV gp120 envelope glycoprotein, in addition to using the tech­niques described above for manually extending the trace from a polyalanine-backbone model derived from a high-resolution homologue, side-chain assignment was aided by negative *B*-­factor sharpening as well as by using the heavy-atom selenium sites and glycosylation sites in conjunction with alignment to the HIV model (Chen *et al.*, 2005 ▶). In addition to using high-resolution substructures as molecular-replacement probes, it has also been possible to use homology models of subunits. This method was successfully used in determining the structure of human factor VIII at 3.98 Å resolution (Ngo *et al.*, 2008 ▶). The presence of side chains in both the previously solved high-resolution subunits and the homology model allowed manual refitting and modelling to generate a complete model that could be effectively refined.

An existing high-resolution or homology model is not always required as a starting point. It is possible to use automated model-building software even at low resolution to generate backbone fragments which can then be used by other automated software to extend and assign the sequence. This was achieved for the structure of human 5-lipoxygenase-activating protein at 4.0 Å resolution, which used a combination of the helix-building module of the *ARP*/*wARP* package (Langer *et al.*, 2008 ▶) and *MIFit* (Ferguson *et al.*, 2007 ▶). The sequence assignment was greatly aided by using the 18 selenomethionine sites and six bound inhibitor molecules as markers.

Sometimes the structural differences between existing high-resolution structures that could potentially be used as either molecular-replacement probes or as manually fitted subunits are too great to be used directly. This was the case for the cocrystal structure Lig1–Lig4 (Dore *et al.*, 2006 ▶), which had a number of significant topology changes and low sequence similarity to its high-resolution homologue XRCC4. To overcome these issues, the structure was traced manually using general topology, alignments and structure prediction gained from knowledge of the homologue. With the lack of side-chain placement from positioned high-resolution sub­units, sequence assignment is much more problematic. Key features in the electron density were identified, in­cluding a glycine–proline–proline 90° turn, a tryptophan located in the middle of a helix supporting a three-stranded sheet identified as a feature from a structural homologue and residues in­volved in the interaction between two subunits; these all acted as starting points for sequence assignment. In this example, side-chain modelling was achieved using the semi-automated real-space search algorithm *RAPPER* (Furnham, Dore *et al.*, 2006 ▶), allowing a number of alternative main-chain/side-chain placements to be ex­plored.

**Figure 3:**
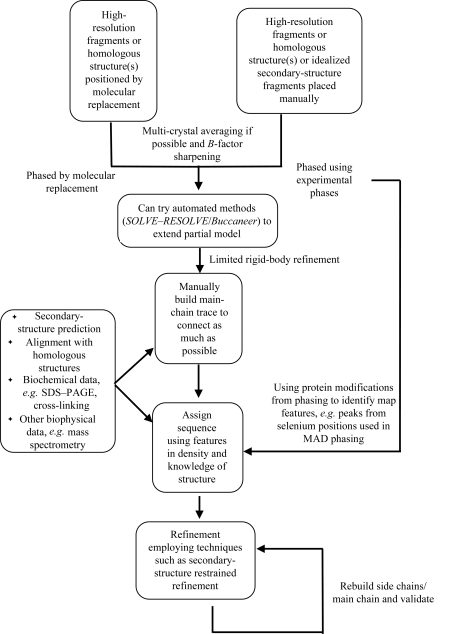
Schematic of a general strategy for low-resolution X-ray crystallographic model generation.

From the different strategies described above, the following action plan for building an atomic model can be employed when presented with low-resolution X-ray crystallographic data. Firstly, all available structural (pre­viously solved fragments and homologous structures) as well as structurally related information (including theor­etical and biochemical data) should be collated together. If related structures exist, they should be located either through use as molecular-replacement (MR) probes, for example using *Phaser* (McCoy *et al.*, 2005 ▶), or if experimental phases have been estimated by manual placement in the electron density using graphical software packages such as *Coot*. Sections of secondary structure can be located in the electron density using fragment libraries and search tools in programs such as *Buccaneer*, *ARP*/*wARP* and *PHENIX* (Terwilliger *et al.*, 2008 ▶). Sections of model can then be connected using automated approaches such as *RAPPER* for smaller loops or by manual extension in *Coot* or *O*. Model building can be informed by secondary-structure predictions based on sequence and also by locating amino acids in the sequence from biochemical data and by binding of heavy metals used in experimental phasing. Sequence placement can also benefit from the association of a particular motif with a usual structural feature. Rebuilding using automated methods such as *RAPPER* or real-space refinement methods as implemented in *Coot* can be used to improve further the model in conjunction with rounds of careful refinement. An overview of this general strategy is shown in Fig. 3 ▶. What is evident from the strategies employed in the past is that an inventive combination of approaches is frequently required in order to interpret successfully the experimental data.

## 4. Interpreting high-resolution electron microscopy electron-density maps

Although X-ray crystallography and NMR spectroscopy remain the methods of choice for studying biomolecular structures to atomic detail, electron microscopy (EM) serves as a complementary tool to study large complexes and macromolecular machines that are difficult to crystallize and are beyond the size threshold for NMR spectroscopy.

Structural interpretation of EM maps generally involves fitting a high-resolution X-ray/NMR structure or a homology model into the map. The model is first docked using programs that perform a global rigid-body search (Volkmann & Hanein, 1999 ▶; Wriggers & Birmanns, 2001 ▶; Wriggers *et al.*, 1999 ▶). The initial fit can then be refined by limiting flexibility to between domains or in connecting loop regions in order to prevent overfitting at low resolution (Chen *et al.*, 2001 ▶, 2003 ▶; Gao *et al.*, 2003 ▶; Topf *et al.*, 2008 ▶). Normal-mode-based methods avoid the need to arbitrarily assign rigid and flexible regions by allowing shifts along the low-frequency normal modes of the molecule (Tama *et al.*, 2004 ▶). Unrealistic distortions are avoided by iterating the procedure and the structure is gradually optimized to fit the density map.

Recent advances in sample preparation and data handling (for a detailed review, see Zhou, 2008 ▶) have lead to the resolution obtainable by EM reaching near-atomic levels. This has prompted the development of new methods that combine traditional model-building techniques taken from crystallo­graphy with new pattern-recognition algorithms (Kong *et al.*, 2004 ▶) suited to sub-nanometre resolution maps. At resolutions of between 4 and 8 Å helices can be identified by their characteristic cylindrical shaped density and β-sheets appear as flat continuous regions of density, although individual strands may not be identified. At resolutions closer to 4 Å, density for bulky side chains may be seen (Chiu *et al.*, 2005 ▶). Visual inspection can be used to identify these features; however, the interpretation can be subjective (Chiu *et al.*, 2002 ▶). Automation has been achieved using *SSEHunter* (Baker *et al.*, 2007 ▶), a feature-extraction program that identifies secondary-structure elements in maps of up to 10 Å resolution. The map is first quantized by designating pseudo-atoms that correspond to regions of high density and then traced using a thinning and pruning algorithm. The skeleton outline gives a simplified geometric representation of the map in which cylindrical shaped density characteristic of helices is represented as a curve and plate-shaped density corresponding to β-sheets is depicted as a surface. α-Helices are identified using a cross-correlation-based exhaustive search between the map and the density of a prototypical helix. The pseudo-atoms are given a combined weighted score based on the skeletal features observed, their relative distance to a high-density voxel in the helix-correlation map, the number and relative geometric positions of neighbouring pseudo-atoms and the aspect ratio of the local density region. Depending upon the score, the pseudo-atoms can then be interactively grouped to represent helices and sheets or an automated procedure can be used.

Once the positions and orientations of secondary-structure elements have been identified, a prototypical helix/strand can be fitted. Although the skeleton can be used as a guide to establish the connections between the secondary structures, branches can occur in regions of ambiguity. Ludtke and coworkers have shown that consensus secondary-structure prediction can be used to assign each C^α^ atom in the helix by mapping the sequences of the predicted helices onto the helices identified in the map based on their lengths and relative position. Connectivity can then be established based on the sequence and the surrounding density. This approach has been successfully used to build a C^α^ trace for the major capsid protein gp7 of epsilon15 virus at 4.5 Å resolution (Jiang *et al.*, 2008 ▶) and GroEL at 4 Å resolution (Ludtke *et al.*, 2008 ▶). β-­Sheets pose a more challenging problem for model building. Although *SSEHunter* can determine position and orientation, differentiating the number and direction of individual strands is difficult. The manually placed C^α^ atoms can then be refined to optimize the fit to the density and idealize hydrogen bonds and dihedral angles within helices and sheets. Although these methods have been developed for EM maps, they could equally be applied to low-resolution X-ray crystallography maps.

Often, some of the helices can be identified and structure-matching programs such as *COSEC* (Mizuguchi & Go, 1995 ▶; Kinoshita *et al.*, 1999 ▶) and *DejaVu* (Kleywegt & Jones, 1997*b* ▶) can be used to probe a library of PDB structures to identify possible homologues based on the relative position and orientation of the helices (Jiang *et al.*, 2001 ▶). Such partial structure-based fold recognition enables homologues to be identified that may have low sequence similarity but share a similar fold. The homologous structure can then be docked and flexibly refined into the map or can aid the model-building process by fitting fragments from structurally/sequentially conserved regions and help establish topology in regions in which loops appear disordered.

## 5. EM resolution and X-ray crystallographic resolution

The method of determining the resolution of an EM map is dependent on the nature of the sample imaged (Chiu *et al.*, 2005 ▶). For two- and three-dimensional crystalline samples and filaments with helical symmetry, resolution relates to the highest peak that can be resolved in diffraction space. For single-particle cryo-EM the data are divided into two sets from which independent reconstructions are calculated and compared at various frequency shells in Fourier space (Leschziner & Nogales, 2007 ▶). Resolution can then be evaluated based on the Fourier shell correlation (FSC; Harauz & Van Heel, 1986 ▶) or the spectral signal-to-noise ratio criterion (Unser *et al.*, 2005 ▶). The more commonly used FSC method ascertains the resolution as the frequency interval at which the two reconstructions have a normalized correlation coefficient equal to a certain threshold, 
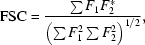
where *F*_1_ and *F*_2_ are the complex structure factors of the two reconstructions and the sum is over all Fourier space voxels contained in a resolution shell.

Generally, a cutoff of 0.5 is used and other cutoff criteria have been proposed (Rosenthal & Henderson, 2003 ▶). It has been argued that the use of fixed-value threshold cannot give a reproducible resolution value. Instead, threshold curves such as the σ-factor curve are more representative and give a more conservative estimate of the resolution (Saxton & Baumeister, 1982 ▶); the 1/2 bit-information threshold curve is calibrated to give a resolution value comparable to the resolution value calculated in X-ray crystallography (van Heel & Schatz, 2005 ▶).

Since the definition of the resolution of an EM map is variable in terms of the criterion chosen to evaluate it, it is thus important that the resolution be validated with the structural details that can be discerned in the map. One might expect that an X-ray map would be more detailed than an EM map at the same resolution; however, a comparison (Cate *et al.*, 1999 ▶) of the X-ray map of *Thermus thermophilus* 70S ribosomal complex obtained at 7.8 Å resolution with the cryo-EM map of the *E. coli* 50S subunit at 7.5 Å resolution (Matadeen *et al.*, 1999 ▶) showed that the visual details observed in the latter were slightly better (van Heel, 2000 ▶).

## 6. Towards automated strategies for model building into low-resolution X-ray data

The latest developments in automated model-building techniques, which allow more indistinct descriptions of the fragments/residues that are used as the basic search models, have extended the resolution at which these programs can effectively generate at least a partial model. These programs include *Buccaneer* (Cowtan, 2006 ▶), *SOLVE*/*RESOLVE* (Terwilliger, 2003 ▶) and the secondary-structure recognition package of *ARP*/*wARP* (Langer *et al.*, 2008 ▶). Other methods, such as *RAPPER*, that combine prior knowledge about a structure, such as secondary structure and sequence, with the experimental data are beginning to emerge for low-resolution X-ray crystallography (Furnham, Dore *et al.*, 2006 ▶). This permits hypotheses and weak assumptions about a structure to be tested. As more automated approaches emerge, it becomes increasingly possible to generate multiple models representing the data. This permits the exploration of the conformational space represented in the data, providing both a measure of the uncertainty in the interpretation of the electron density and the temporal and spatial heterogeneity of the structure (Furnham, Blundell *et al.*, 2006 ▶).

Developments in low-resolution X-ray crystallographic structure modelling are beginning to mirror some of the recent developments in model generation for EM. As attempts are made to understand the mechanisms of complete molecular machines, there is a need to integrate more diverse structural data. In the determination of the architecture of the nuclear pore complex, spatial restraints derived from EM maps together with proteomics experiments and biophysical studies such as ultracentrifugation and affinity purification were used to restrain relative positions of individual protein components in a molecular simulation to generate ensembles of possible architectures for the complex (Alber *et al.*, 2007 ▶). As many of the same challenges in providing high-confidence models of macromolecular structures and assemblies are shared by both EM and low-resolution X-ray crystallography, it is likely that many of the methods will be combined and new strategies developed to provide more automated techniques for model construction.
